# Significant EHR Feature-Driven T2D Inference: Predictive Machine Learning and Networks

**DOI:** 10.3389/fdata.2019.00030

**Published:** 2019-09-27

**Authors:** Nicolo' Preo, Enrico Capobianco

**Affiliations:** ^1^Bip xScience, Milan, Italy; ^2^Center for Computational Science, University of Miami, Miami, FL, United States

**Keywords:** electronic health records, type 2 diabetes mellitus, feature selection, network inference, communities, patient stratification

## Abstract

**Background:** Electronic health records (EHR) play an important role for the redefinition of phenotypes in view of the wealth and heterogeneity of information now available from disparate data sources. A recent cross-sectional retrospective study has described the potential of EHR toward type 2 diabetes mellitus (T2D) screening when *ad hoc* models are used. About 10,000 US patients have been analyzed through a variety of inference techniques applied to all records with a variable degree of completeness. The analyses conducted in the reference study have indicated that EHR phenotypes significantly improved T2D detection.

**Methods:** With these US patients and the T2D data evidenced in the above study, we propose an integrative inference approach that leverages the prediction power of EHR features selected by two well-known methods, Random Forests and Lasso. The goal is 2-fold: reducing the Big Data redundancies potentially harmful to the predictive learning task and exploiting the interconnectivity of EHR features. A mutual information (MI) network is the inference tool used to identify communities useful to prioritize significant T2D features underlying the similarity between patients.

**Results:** Endowed with a different degree of granularity, the communities detected after the application of both methods were centered especially on T2D comorbidities and risk factors. As such, they appear very relevant for assessment of two main issues, T2D disease burden, and prevention.

**Conclusions:** Our analytical approach offers a solution for managing the EHR scale factor in a complex disease context. EHR are rich sources of phenotypic diversity through which novel stratifications of patients are expected. To enable these results, both pre-screening of variables and calibration of risk prediction methods become necessary steps in EHR analyses. We have presented networks identifying major T2D communities. The specific significance assigned to comorbidities and risk factors in relation to T2D can be inferred with accuracy from just a suitably reduced number of EHR features.

## Introduction

Diabetes is a disorder traditionally subdivided into two types. Type 2 diabetes (T2D) is a chronic condition that affects how our body metabolizes sugar or glucose, inducing either resistance to the effects of insulin, or lack of its production in a way sufficient to maintain normal glucose levels. No cure exists for such disorder affecting populations that include adults as well as children. Control of body weight, diet, and exercise can help T2D management, complementing (or as an alternative to) medications or insulin therapy.

T2D is of interest to this work. The classification of diabetes depends primarily on age at onset and the presence or absence of conditions such as obesity, metabolic syndrome, insulin deficiency, and others. Several mechanisms can lead to diabetes, and these can be modified by genetic, lifestyle, and environmental factors. Clearly, all such factors make T2D a very heterogeneous disease, one for which many types of data should be analyzed for achieving superior precision of diagnoses and therapies. The identification first of the informative features and patterns within these complex “Big Data” sets and then of the linkages to outcome data may yield valuable insights into risk factors, diabetes history, and comorbidities, in turn advancing both prevention and management of the disease from a Precision Medicine (PM) perspective (see, for instance, Capobianco, 2017; Fitipaldi et al., 2018; Xie et al., 2018; Prasad and Groop, 2019).

PM springs from a variety of new technologies and aims at a patient's approach based on personalization. Therefore, PM explores the distinct characteristics in individuals that make their disease signatures or risk profiles possibly unique. This complexity involves the acts of first collecting widespread information specific to the individual and then streamlining data-driven processes subject to treatment by automated rules. A domain of support to PM data analytics is electronic health records (EHR). EHR represent complex heterogeneous information systems that call for algorithmic approaches in order to quantify their saliency and the related uncertainty. Once these two properties are suitably assessed, one of the most valuable roles that EHR may play is to generate new disease phenotypes, leading to novel classifications, and taxonomies (Pendergrass and Crawford, 2019). PM approaches beyond one-size-fits-all models respond to the challenge of deciphering the interactions between EHR components. These have value especially in predictive terms, knowing that prediction represents a crucial outcome of data analytical tools. Additionally, there is translational value potentially achievable when disease patterns are discovered before the appearance of patient's symptoms, or when risk and/or outcome profiles are evaluated in patient populations for stratification purposes.

Our work re-analyzes EHR referred to US patients that were previously investigated to assess whether the associated phenotypic features would potentially improve T2D screening. The examined conditions involved a variety of possible data models and various levels of data incompleteness (Anderson et al., 2016). The application of a multilevel approach delivered indications that EHR phenotyping is valuable due to the ability of identifying patients who are seen as candidate or not for further screening according to classical practices (lab work, etc.). This result is relevant in predictive terms, because when applied to yet undiagnosed individuals, it would allow detection with accuracy superior to other types of pre-screenings. However, a current challenge is to assess what parts of EHR are indeed needed to outperform predictive learning algorithms designed to run over other data types (i.e., non-EHR studies). In general statistical terms, this problem has some relevance for the task of computing sufficient statistics, say T. Certain models are based on probability distributions representative of the reference populations characterized by features that may be parameterized within the models. EHR are typically seen as a collection of large samples for which sufficient statistics by definition would be those containing all the sample information. Clearly, EHR patient heterogeneity cannot be categorized or reconciled under one distributional model. Aiming to mitigate the complexity inherent in EHR heterogeneity, networks are the approach here chosen to infer EHR salient features and their latent interconnectivity.

## Methods

### Data Curation and Mining

The reference dataset consists of a matrix with 300 variables and 9,948 medical records, of which 165 report on individuals suffering from T1D. This last fraction is excluded from further analyses, which are instead centered on T2D (about 20% of the reference population, see the Supplementary Table 1). Most variables are dichotomic, while other discrete variables have been dichotomized, i.e., *MetabolicDX, Chantix*, and *Seroquel*. Three categories appear for the smoking variable (never, no longer, active) with many missing values (5,547), and is therefore excluded from further analyses (in any case verified as non-influential for the results). Blood pressure (diastolic and systolic) has been excluded too, as well as a few other variables (*TotalRiskFactors, TotalDiagnosesGeneral, TotalAcute, TotalMeds, TotalLabs, TotalAbnormalLabs*, etc.) for reasons of duplications and redundancies that increase the risk of collinearity. *BMI* was categorized according to four weight ranges: 0–18.49 (underweight), 18.5–4.99 (normal weight), 25–29.99 (overweight), and >30 (obese). Age includes individuals of age > 18 (discrete variable). After the initial checkup, the final matrix counts 8,482 medical records and 286 variables. The population is 43% men, of which 21% have T2D, and ranges between 18 and 76 years of age. The two most represented classes of variables, both dichotomic, are centered on diagnoses, and medications.

### Data Analysis

As a first step toward (a) the identification of a set of most predictive variables and (b) computing their interconnectivity, feature saliency was considered. Many existing methods can treat and prioritize variables for the scopes of feature selection. We selected two highly popular methods, Random Forest (RF) (Ho, 1995, 1998; Hastie et al., 2009; Breiman, 2011), and LASSO (LA) (Santosa and Symes, 1986; Tibshirani, 1996, 1997).

Specifically:

- RF is based on combining decision trees to predict outcomes. The random extraction of variables at every tree node enables a best split, i.e., the one that minimizes a loss function [deviation, Gini index (Gini, 1909, 1936), etc.].
  $$\begin{array}{l} \text{Deviance}=-2\sum_{i=1}^{n}\left\{y_{i}\log\hat{P_{i}}+\left(1-y_{i}\right)\log\left(1-\hat{P_{i}}\right)\right\},\\ \text{ with }{\hat{P}}_{j}=\frac{1}{n_{j}}\sum_{i\in R_{j}}\text{I}\left(y_{i}=1\right),R_{j}\in {\text{R}}^{\text{m}}.\\ \;\text{Gini}=\sum_{k=0,1}P_{jk}\left(1-P_{jk}\right) \end{array}$$
  The commonly used Gini index is computed for every variable and measures at each split the entropy reduction contributed by the variable selected for tree splitting. A 10-fold cross-validation was adopted over a dataset with 70% of the population to estimate the parameters.

Prediction is also associated to the relevance assigned to the variables, one being measured by the mean decrease accuracy (MDA). Since this is a measure of accuracy over the excluded observations, i.e., those not extracted to decide over the tree split, the result that is provided is an out-of-bag classification error at each split. Once this is done recursively by permuting the values of each variable involved at every split and computing an average (cross-splits) difference between pre- and post-permutation misclassification errors, an estimate is obtained for the influence that each variable has over the model prediction power.

$$\text{Misclassification Error}=\sum_{j=1}^{J}\frac{1}{n_{j}}\sum_{i\in R_{j}}I\left(y_{i}\neq C\left(\hat{p}(x_{i})\right)\right)$$

- LA is based on regression, and shrinkage (hard, reduction to 0) is enabled over the coefficients that are found as the least informative, thus leaving only the most relevant variables associated to the surviving coefficients. This is a regularized estimator aimed to trade off distortion by reduced variance. The minimized function (below reported in Lagrangian form) is:
  $$\begin{array}{l} \sum_{i=1}^{n}{\left(y_{i}-x_{i}^{T}\beta \right)}^{2}\text{subject to}\sum_{j=1}^{p-1}\left|\beta_{j}\right|\;\leq \;s\\ {\left\|y-X\beta \right\|}^{2}+\lambda \sum_{J=1}^{p-1}\left|\beta_{j}\right|\text{ with }\lambda \text{ tuning parameter}. \end{array}$$

### Networks

Given the selected variables, our inference method is centered on networks. Nodes (variables) and links (relationships among variables) established interconnectivity patterns leading to the identification of communities, i.e., modules showing component nodes more significantly connected than random and/or non-component nodes. Node size was computed in agreement with relevance of the reference variables, and a logistic regression was run over the variables that were selected by either RF or LA. We therefore assumed that beyond the simple lists of variables selected by RF and LA, networks add information on the linkages between these variables. This is very useful to assess a variety of T2D characteristics and their role for both diagnostics and therapeutic scopes. Utility also covers T2D prevention aspects, i.e., a valuable context for which the prediction power of the variables here analyzed should be optimized.

With reference to the T2D sub-population, the links were established with width dependent on the most robust metric, i.e., mutual information (MI) (Shannon and Weaver, 1949; Gray, 1990; Cover and Thomas, 1991).

$$\text{MI}=\sum_{y\in Y}\sum_{x\in X}p\left(x,y\right)\log\frac{p\left(x,y\right)}{p\left(x\right)p(y)}$$

For independent variables, this measure is minimized. The association with significance can then be established via permutations (Monte Carlo). Once these were done, robust communities were computed via a known fast-greedy hierarchical bottom-up method. Iterations of the algorithm maximize the modularity achieved incrementally, and with no use of tuning parameters. Greedy learning always implies that global optimization is to be replaced by only local optimization, as this is the only goal compatible with the nature of the search in the solution space.

## Results

**Figure 1:**
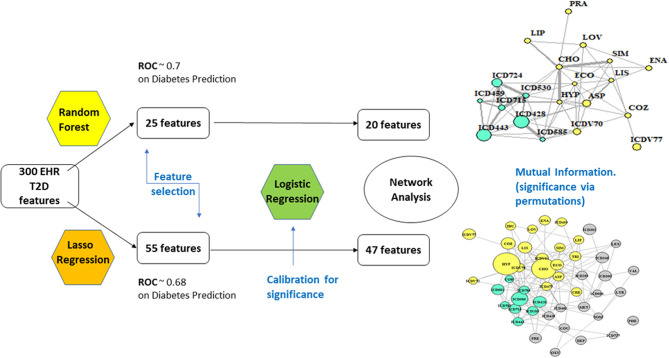
Analytical flowchart.

Figure 1 offers a graphical description of our methodological pipeline.

### Feature Selection

- RF: With 67% accuracy and 70.4% of ROC (receiver operating characteristic) performance metric, the results are considered of good quality. ROC analysis provides tools to select optimal models and assess the performance of classifiers over their entire operating ranges. Boxplots have then been computed over the distributions of values obtained by the two measures, MDA and Gini. The variables selected were 25, based on +1.58 3QR/sqrt(n). They depend on asymptotic normality of the median and similar sample sizes for the two medians being compared, which makes results insensitive to the underlying sample distributions. An ~95% confidence interval for the difference between medians is thus obtained.
- LA: the strength of the shrinkage was obtained by a single parameter through 10-fold cross-validation over 70% of the population, testing then over the residual 30%. Results were similar to RF, only slightly worse (accuracy = 65%, precision = 35.7%, recall = 70.64%, ROC performance = 68%). The selected variables remained at this point 55.

#### Networks

Nodes have size proportional to the *z*-score computed from the estimates obtained for coefficients and their standard errors in the logistic regression step (Cox, 1958; Walker and Duncan, 1967). RF yielded a network of highly significant 20 variables and LA a network of 47.

**Figure 2:**
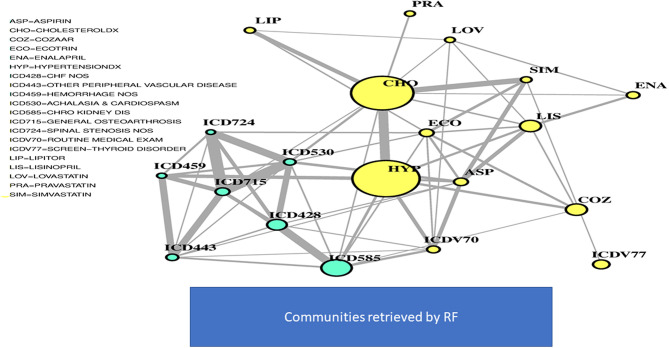
RF-driven T2D network. Global connectivity map.

**Figure 3:**
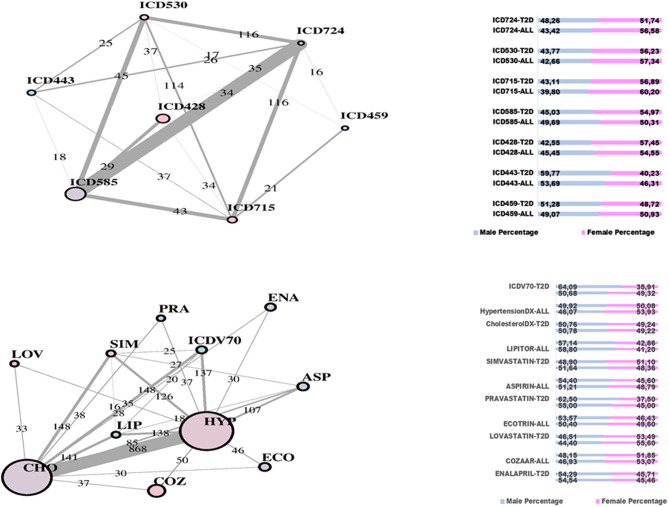
RF-driven T2D network communities. Edge numbers refer to patients sharing morbidities **(Top)** and risk factors **(Bottom)**. The communities are edge-constrained: at least 15 patients are reported. The variable “sex” colors the nodes to indicate prevalence of males (green) or females (pink).

In Figure 2, two communities are emphasized from the RF network. The “green community” was enriched by variables defining T2D comorbidities. In order of descending node size, we have: chronic kidney disease (ICD585), congestive heart failure (ICD428), general osteoarthrosis (ICD715), gastroesophageal reflux (ICD530), vascular disorders (ICD443), disease of the circulatory system (ICD459), and spinal stenosis (ICD724). The “yellow community” was enriched by variables defining risk factors. Apart from the two largest nodes, Hypercholesterolemia (CHO) and Hypertension (HYP), the other nodes of greatest relevance are correspondingly referred to LIPITOR, PRAVASTATIN, and SIMVASTATIN for cholesterol control, and hypertension medications like COZAAR and ENALAPRIL. Then, ECOTRIN and LOVASTATIN whose indications are for contrasting RA and CVD, respectively, plus ASPIRIN. Additional details are displayed in Figure 3 with edge numbers referring to communication among variables (i.e., number of patients sharing the nodes) and also gender-related prevalence (rightmost barplots).

**Figure 4:**
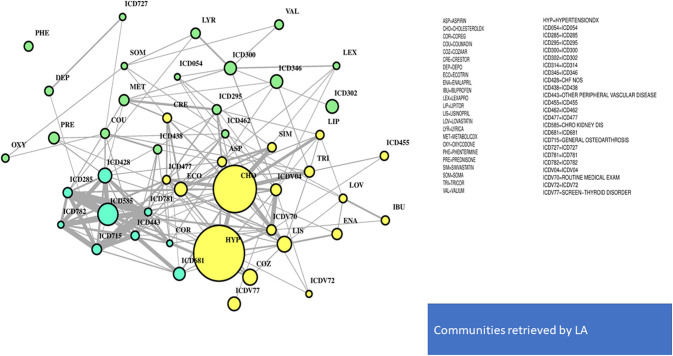
LA-driven T2D network. Global connectivity map.

**Figure 5:**
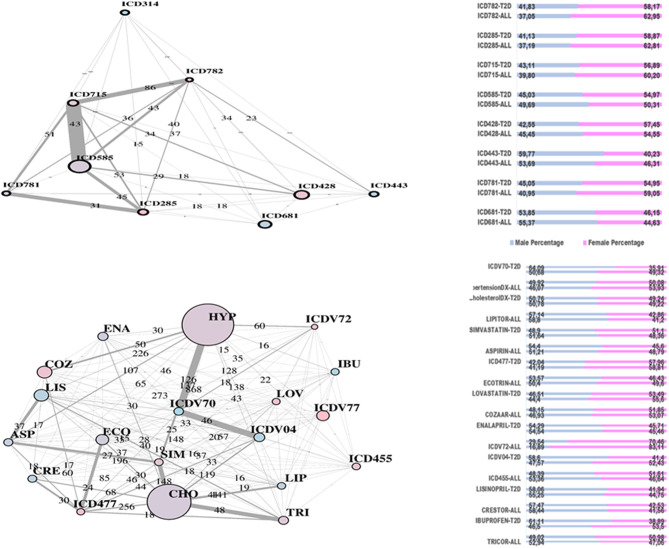
LA-driven T2D network and communities. Edge numbers refer to patients sharing morbidities **(Top)** and risk factors **(Bottom)**. The communities are edge-constrained: at least 15 patients are reported. The variable “sex” colors the nodes to indicate prevalence of males (green) or females (pink).

In Figure 4, the global network map obtained from LA appears more redundant than the RF one. In part, this is induced by LA selecting almost twice the number of significant variables obtained with RF. In particular, Figure 5 (top plot) shows the first identified community in the LA network largely overlapping with the comorbidity identified in the RF network for a few variables, namely, chronic kidney disease (ICD585), congestive heart failure (ICD428), general osteoarthrosis (ICD715), and vascular disorders (ICD443). The order of significance is also preserved, except for the presence of ICD681 (toe and finger abscess). Other variables referred to symptoms of disorders related to anemia (ICD285), nervous system (ICD781), and skin (ICD782).

**Figure 6:**
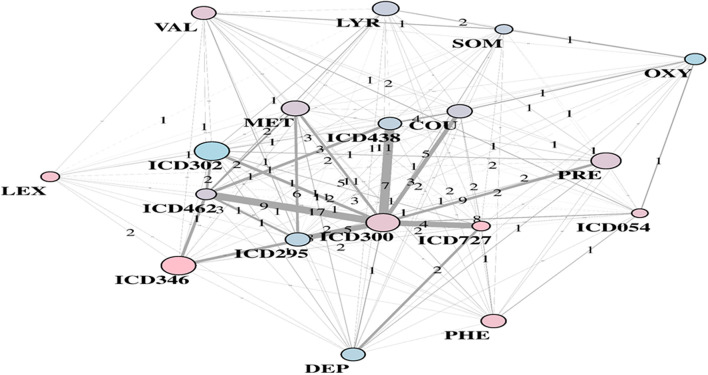
LA-driven T2D supplemental community, with constrained links and central role of hub ICD300.

Substantial redundancy also appears from the other identified community of risk factors (bottom plot of Figure 5), but with cholesterol and hypertension remaining the main hubs. An increased number of medications appears compared to the corresponding RF yellow community. Another specific community is identified in the LA network (see Figure 6), and it shows disorders and drugs with a certain prevalence assigned to anxiety (the hub ICD300 is central, but also drugs such as Valium and Lexapro) and other neurological conditions (depression, cognitive deficit, schizophrenia, etc.).

Overall, our results indicate that the redundancy observed with LA (compared to RF) is well reflected into the communities. This effect is independent of the presence of bounds over the number of patients sharing nodes, which here were established to facilitate both interpretation and visualization of the dynamics. While methods' sensitivity exists (to a certain extent) with respect to the selection of variables, we stress the fact that the major communities extracted in both scenarios are enriching risk factors and comorbidities, two key aspects in T2D study. This basically means that community structure in networks may undergo different temporal events linked to T2D and the associated dynamic transitions through a few predictive features significantly reducing the complexity and redundancy of Big Data.

## Discussion

Disease maps can be generally defined as global networks offering representation of observable interconnected factors and variables underlying the disease mechanisms. Conducting inference over such mechanisms requires the identification of any possible relationships between measurable variables. Although some of these relationships may remain latent, either because unobservable or not measurable in their characterizing variables, the accuracy of disease maps can be quite substantial to provide insights. In the presence of complex diseases, an additional layer of complexity comes from the association of multiple conditions affecting the same patient, especially during disease progression. This association generates composite disease maps in which the physiological and pathological mechanisms one aims to infer partially overlap. Clearly enough, the best use of these maps is for predictive inference, and indeed our predictive networks (and their embedded communities) use massive amounts of data to learn from history and identify the domains most suitable to predict events characterizing T2D.

More specifically, our methodological approach identifies from EHR a few specific drivers ensuring predictability, generalizability, and reproducibility of results based on the observed T2D patient population: (a) variables prioritized by two machine learning algorithms; (b) networks as tools to conduct inference over interconnected variables explaining T2D in the EHR; and (c) significant interaction dynamics represented by the identified communities. Knowing the importance of consensus between methods, we showed the role played by network communities in reconciling the variability observed among selected variables. Communities absorb diversity among individuals who share major features (risk factors, comorbid conditions) underlying T2D, and suggest an effective approach to patient stratification. The choice between methods selecting such features has been limited to a couple of examples, and of course an improved association with T2D may be expected when more methods are compared (Bernardini et al., 2019). Generalization of our approach to other disease contexts is immediate and also allows reproducibility in terms of specific disease aspects here identified with comorbidities and risk factors.

In general, PM promises to change disease treatment and prevention by taking into account individual variability expressed in many ways, from genes to environmental marks, to diet and lifestyle for each person. This is exactly the type of data we may expect to find in EHR and relevant to T2D too. Previous work (Richesson et al., 2013; Spratt et al., 2016) assessed EHR phenotypes against gold standard diagnostic ADA criteria for T2D with reference to a dataset comprising 173,503 patients from the Duke University Health System. Phenotype definition in these studies was considered a very important aspect of the analysis, but more important is to assess the impact of EHR in terms of predictive learning. Our results indicate that salient predictive features are well-recapitulated by EHR. The results in Anderson et al. (2016) showed overlap with those evidenced by our communities with reference to positive associations with T2D. Examples are provided by the significance of hypertension together with medical tests, plus a number of other non-significant entries such as enalapril, pravastatin, lisinopril, simvastatin, heart failure, and chronic kidney disease.

It is worth also questioning EHR in the presence of contradictory results. Among the unexpected factors negatively and significantly associated with T2D diagnosis, there are diseases of the esophagus and other bone disorders (ICD733). Other surprising variables were emphasized in Anderson et al. (2016), especially with reference to sexual and gender identity disorders (ICD302), or also chlamydial infections generating multiple associated conditions. In our study, the LA community shown in Figure 6 included ICD302 (showing male prevalence and quite low in link counts) as connected to the hub ICD300, centered on anxiety. This might indicate that a group of conditions revealed as significantly connected may have only indirect association with T2D, due to general use of antidepressant drugs or to comorbid conditions.

We remind that several of the studies examined here have inherent limitations, such as the fact that no prognostic value was assigned to factors aimed to emphasize causal associations, one of the reasons for excluding the consideration of specific factors, for instance, those intertwining between T2D and CVD diagnoses. Space is therefore available for future revision of model performance as defined in our work to possibly encompass other subtle T2D features. Shedding further light on causality will require application of readapted models, or at least models revised in their performance criteria.

Potential for such extensions has already appeared from our current work. We considered the results from other large-scale T2D studies for comparative evaluation of the detected comorbidities. One such study (Razavian et al., 2015) is the largest (despite geographically concentrated) available based on about 4 million insurance records referred to beneficiaries who then matched T2D inclusion criteria in a fraction of about 800,000 individuals. Of these, about 19,000 (14,000) revealed T2D cases along 2009–2011 (2010–2012), with about 42,000 variables used to summarize the medical status and reduced to about 1,000 (800) significant ones. At a methodological level, this study not only distinguished between inclusion of small to broad feature sets but also considered the impact of temporal trends. Thus, the purpose was primarily to find a risk prediction model for T2D valid at the population level and secondarily to rank such factors according to their power to early prediction of disease onset, and the top predictive variables for immediate vs. delayed T2D onset were presented. Interestingly, among the variables with 1-year predictive value, there is esophageal reflux, appearing also in our green community of Figure 2 as well as emphasized by Anderson et al. Despite diabetes being one of those conditions associated with many others, Caughey et al. (2010) indicated CVD as the prevalent morbidity, determining a high number of prescriptions, and gastroesophageal reflux disease was also shown in such study to be prevalent.

Another example is associated to the study proposed by Li et al. (2015) and based on EHR and genotype data of about 11,000 outpatients from diverse communities in the NY area. A topology-based approach was developed to infer a patient–patient similarity network. Two major clusters were identified, enriching a variety of morbidities, and once a T2D phenotype algorithm is defined, a few subnetworks were considered as T2D clusters. From the examination of such clusters, we find that among the significant variables in each group, heart-related diseases, and other circulatory disorders appear in all groups and specifically in one together with kidney-related diseases. In this case, compared to our coarser analysis, both heart-, and kidney-related conditions appear enriched also for significant phenotypes with disease-genetic variant.

Another interesting phenotype also appeared significant in one of the T2D clusters from the above study, osteoporosis. This linkage also emerges from our analysis, as there are two variables related to bone problems. Notably, this feature was investigated in a review (Duclos, 2016) elucidating the pathophysiology of the association with T2D and obesity. While the latter is responsible for excess mechanical load over cartilage, another aspect refers to adipose tissue effect and thus local-to-systemic inflammation that, in diabetics, is amplified by insulin resistance, further damaging cartilage, bone, and synovial tissue. Additional findings about RA are emerging, from which it is known that the use of steroids can increase T2D risk, as well as osteoarthritis, osteoporosis, and back pain, all found significant in patients from a Danish study with more than 9000 cases with diabetes (for details, see Molsted et al., 2018).

Continuing with the synergies between results, and by looking at the risk factors evidenced in the yellow community with the RF network, cholesterol and hypertension are present among the predictive variables in Razavian et al. (2015) with reference to the first 3-years period, while antiarthritic medications appear with reference to both temporal periods. In the other study previously recalled (Li et al., 2015), the condition hypercholestorelemia appears in the first specific T2D cluster together with heart and kidney significant phenotypes (and enriched also by disease-genetic variant).

## Conclusions

A number of other specific considerations related to the results emerging from our analysis deserve to be mentioned with reference to their relationships with other studies, and the limited treatment reserved to them at this stage represents a motivation for future investigations. When considering T2D drugs, for instance, it would be important to emphasize potential conflicts emerging on the presence of other conditions, as it is typical in patients exposed to polypharmacy (Franchini et al., 2015). Conditions such as RA are considered of relevance for T2D with reference to treatment by corticosteroids. Also, some discordant health conditions like back pain tend to not be considered in the diabetes guidelines because of their aspecificity.

Remaining confident that a generalization of our approach to other complex diseases is straightforward, we stress the fact that the key strategy that we have illustrated with the T2D data combines EHR processing via feature selection with post-processing via networks. The latter were shown to yield community-driven inference tools delivering variable prioritization in support of patient stratification. The role of comorbidities and risk factors emerged significantly, suggesting that their underlying features are retrievable from EHR and could be well-contextualized by network communities.

Future challenges include cross-referencing these results with newly available T2D datasets for establishing consistency and robustness as well as generalizability to other complex disease contexts. Comparative evaluations between the proposed methodological approaches and other novel ones will likely allow in the near future more accurate determination of significance for some T2D-specific variables (Bernardini et al., 2019). More in general, a reduction of the Big Data uncertainty related to some detected phenotypes and their disease associations might be expected from the implementation of automatic learning strategies, such as deep learning (see, for instance, Osmani et al., 2018; Xiao et al., 2018).

## Author Contributions

All authors listed have made a substantial, direct and intellectual contribution to the work, and approved it for publication.

### Conflict of Interest Statement

NP was employed by the company Bip. The remaining author declares that the research was conducted in the absence of any commercial or financial relationships that could be construed as a potential conflict of interest.

## Supplementary Material

Supplementary Table 1 Data summaries.
